# A Passive Tracking System Based on Geometric Constraints in Adaptive Wireless Sensor Networks †

**DOI:** 10.3390/s18103276

**Published:** 2018-09-29

**Authors:** Biao Zhou, Deockhyeon Ahn, Jungpyo Lee, Chao Sun, Sabbir Ahmed, Youngok Kim

**Affiliations:** 1 School of Internet of Things Engineering, Jiangnan University; Wuxi 214122, China; zhoubiao@jiangnan.edu.cn (B.Z.); Asabbir@jiangnan.edu.cn (S.A.); 2 Department of Electronic Engineering, Kwangwoon University, Seoul 01897, Korea; ejrgus456@gmail.com (D.A.); jp.lee.life@gmail.com (J.L.); sunchao2601@gmail.com (C.S.)

**Keywords:** passive tracking, geometric constraint, multiple targets, radio frequency tomography, adaptive networking

## Abstract

Target tracking technologies in wireless sensor network (WSNs) environments fall into two categories: active and passive schemes. Unlike with the active positioning schemes, in which the targets are required to hold cooperative devices, the research on passive tracking, i.e., tracking device-free targets, has recently showed promise. In the WSN, device-free targets can be tracked by sensing radio frequency tomography (RFT) on the line-of-sight links (LOSLs). In this paper, we propose a passive tracking scheme exploiting both adaptive-networking LOSL webs and geometric constraint methodology for tracking single targets, as well as multiple targets. Regarding fundamental knowledge, we firstly explore the spatial diversity technique for RFT detection in realistic situations. Then, we analyze the power consumption of the WSN and propose an adaptive networking scheme for the purpose of energy conservation. Instead of maintaining a fixed LOSL density, the proposed scheme can adaptively adjust the networking level to save energy while guaranteeing tracking accuracy. The effectiveness of the proposed scheme is evaluated with computer simulations. According to the results, it is observed that the proposed scheme can sufficiently reduce power consumption, while providing qualified tracking performance.

## 1. Introduction

In traditional active tracking schemes, the target has to hold cooperative devices for being tracked, such as RFID tags or wireless transceivers, to name a few. However, such tracking schemes have a limitation in specific scenarios; for example, criminals and intruders try their best to avoid carrying any devices for the system to track them, or the device of an uninvited guest may be unable to provide necessary information for the tracking work; moreover, the elderly and children are incapable of effectively operating their wireless device. Targets in such scenarios are called device-free targets. Tracking device-free targets is termed passive tracking or device-free localization (DFL) [1,2].

In wireless sensor networks (WSNs), the received signal strength (RSS) between pairs of wireless nodes is easy to measure when data packages are being delivered between them. The RSS is generally the most practical information for localization works, thanks to its merits of ubiquity and ease of accessibility [3]. Generally, in the scenario of the passive tracking schemes based on RSS information, all the wireless sensor nodes are distributed with known and fixed coordinates in advance and monitor the area of interest. When the device-free target (including intruders, uninvited guests, etc.) walks into the area of interest, it will cause RSS fluctuation between pairs of wireless sensor nodes. We generally define the RSS fluctuation caused by the device-free target as radio frequency tomography (RFT) phenomenon [4]. Actually, recent research and experiments indicate that the RSS fluctuant can be easily detected when a mobile target walks across a line-of-sight link (LOSL) between pairs of wireless nodes [5,6,7,8]. Taking advantage of the RFT phenomenon, a passive indoor tracking scheme is proposed to find the crossing point (CP) on the LOSLs using geometrical the constraint method in reference [5]. Essentially, with the available topology information of the wireless node distribution, the trajectory of the device-free target can be uniquely identified by fusing the triggering sequence of the LOSLs into the geometric constraint strategy.

The performed experiment in reference [5] is under a relatively incomplete indoor environment, and the performance evaluation has not been sufficiently analyzed. Thus, in reference [6], the authors further investigated the performance of the schemes based on the geometric constraint method under various trajectories, node densities, and noisy topologies, by using the practical experiments and simulations.

However, a common drawback of references [5,6] is that their implementations keep all wireless nodes active to form a constant LOSL topology, even when no target appears or the demand of tracking accuracy is not so high, which is insufficient from the perspective of energy efficiency. It is well known that power consumption is a serious challenge in the WSN environment; hence, the dynamic networking mechanism should be considered in the tracking system for energy conservation purposes. Moreover, the issue of tracking multiple targets has been neglected, which is an essential problem in DFL scenarios [2,4,7]. In addition to that, the RFT phenomenon, which is the foundation knowledge, should be further investigated to guarantee the feasibility of the passive tracking scheme based on the geometric constraint methodology.

In this paper, we will pay attention to the energy consumption of the passive tracking system, which was partly introduced in reference [9]. We build a power consumption model for the RFT-based tracking systems in WSN environment. Then, an adaptive networking scheme is proposed for energy saving while guaranteeing the tracking accuracy. Specifically, the new proposed scheme can adaptively adjust the adopted node density according to different situations; as such, the system can adaptively increase the level of the LOSL web to a higher level to get a more accurate trajectory of the target, or decrease the level of the LOSL web for energy saving when accuracy is undemanding.

In general, the contribution of this paper can be summarized as follows:

(1) Using the foundation of the passive tracking schemes based on geometric constraint methodology, we demonstrate the feasibility of identifying RFT phenomenon by utilizing the spatial diversity receiving technique;
(2) To extend the application of the passive tracking schemes based on the geometric constraint method, a method for multiple device-free targets tracking is presented;
(3) We first build a quantifiable power consumption model for RSS-based passive tracking WSNs, and an adaptive networking scheme is proposed with the mechanism of the tracking performance adjustment, as well as energy conservation.

The rest of the paper is organized as follows. In following Section 2, state-of-the-art, RFT-based passive tracking schemes are reviewed. Then, methods of RFT detection of the individual LOSLs are introduced in Section 3. In Section 4, passive tracking schemes for single target tracking, as well as multi-target tracking based on geometric constraints, are presented in detail. The power consumption model of specific passive tracking scenarios is analyzed in Section 5. In Section 6, the adaptive wireless networking scheme is proposed. The passive tracking performance and the power consumption of the proposed scheme are evaluated with computer simulations under different levels of wireless network in Section 7. Finally, our concluding remarks are summarized in Section 8.

The clarification is made as follows: L(X,Y) or L(i) means the LOSL between nodes X and Y or i-th-triggered LOSL; S(X,Y) or S(i) presents the segment between points X and Y or the highest probability segment when *i*-th-triggered LOSL is detected; the intersection of straight lines L1 and L2 is denoted by L1∩L2. R(A,B,⋯,Z) or R(i) means the region represented by the points A,B,⋯,Z or the located region after *i*-th-triggered LOSL is detected.

## 2. Related Works

Recently, the passive tracking scheme adopting the RFT has been addressed as an emerging issue. Among existing passive tracking schemes, the solution based on the radio tomography imaging (RTI) is a popular trend [8,9,10,11,12,13]. Typical RTI-based work is carried out by Wilson et al. in [8,11,13]. Reference [8] presented a linear model for using RSS measurements to obtain images of moving objects. The authors also provided a noise model based on real measurements of a deployed RTI system. Mean-squared error (MSE) bounds on image accuracy are derived, which are used to calculate the accuracy of an RTI system for a given node topology. In Reference [11], they extended their work by presenting a statistical model relating RSS variance to spatial locations of movement and used it as a framework for the estimation of a motion image. From the motion image, the Kalman filter is applied to recursively track the coordinates of a moving target. Above, two RTI methods are both focused on the dynamic targets. To extend the application scenario, in reference [13], they introduced some measurement-based statistical models that can be used to estimate the locations of both moving and stationary people using RSS measurement in wireless networks. Similar works can be referred to in reference [14], in which Viani et al. divided the domain of interest into square regions and used support vector machine (SVM) to train the a priori relationship of target’s location and RSS measurements. At the testing phase, then, the posteriori probability is used to locate the target in a specific region by the fusing the RSS measurements of all LOSLs and the priori relationship.

Besides the global RSS imaging, another trend is applying RSS measurement to the individual LOSLs. However, it is common knowledge that RSS measurement is extremely fragile due to the wireless environment; hence, in reference [15], Talampsa et al. introduced a multichannel geometric filter (MCGF) algorithm by using LOSL fade level and RSS variance on different frequency channels to detect the target-affected LOSL. Then, they use a weighted mean derived from the static fade levels of the target-affected links to generate a location estimated from their intersection points, giving larger weights to intersection points of predominantly anti-fade links. Another LOSL-based tracking scheme is introduced by Wang et al. in reference [16]. They utilized not only the observation information of the shadowed LOSLs but also the prior information involved in the previous estimations and the constraint information involved in the non-shadowed links, which ensure its robust location estimation performance. Their approach represents the prior information, observation, and posterior information with grid maps, and can be implemented with a series of simple grid multiplication and addition operations, which makes it a lightweight scheme suitable for hardware-limited applications. 

Some researchers pay attention to outlier LOSL rejection using probabilistic approaches, rather than improving the RFT detection of the LOSLs. An example is presented in [17], in which Xiao et al. introduce a novel nonlinear optimization approach with outlier link rejection (NOOLR) for RSS-based DFL that consists of three key strategies, including (1) affected link identification using differential RSS detection; (2) outlier link rejection via geometrical positional relationships among links; (3) target location estimation by formulating and solving a nonlinear optimization problem. Experimental results demonstrate that NOOLR is robust to the fluctuation of wireless signals with superior localization accuracy compared with the RTI-based approach. Another particular scheme is introduced by Mao et al. in [18]. They presented a tracking system named “iLight” using optical sensor nodes and one base station; they introduced probabilistic tracking strategies to track both single and multiple targets. The experimental results show that the iLight scheme is not only able to compute the moving trajectories of targets efficiently, but it is also able to study the properties of moving targets (e.g., height). Although they did not use the RFT, the methodology is suggestive for the RFT schemes and worth reviewing here.

However, above schemes all rely on high-density wireless nodes to guarantee tracking accuracy and neglected the power consumption issue. Here, in this paper, we specifically focus on the CP estimation of individual LOSLs and apply the methodology of geometric constraints to track device-free targets by using the information of the WSN topology.

## 3. The RFT Identification

Before applying the geometric constraint, the first issue that should be addressed is whether the LOSL can perfectly sense the RFT phenomenon and get “triggered” once a target is passing through it, considering the RSS is so fickle to be predicted due to the multipath effect. Actually, several methods may help here:

(1) The first method is by using the diversity reception techniques. For example, the frequency diversity is proven to be efficient for detecting the appearance of the device-free target in reference [15], and spatial diversity is also a well-known technique to overcome the multipath effect [19,20].
(2) The second way is to increase the operating radio signal frequency is to weaken the diffraction ability of signal propagation, such as the emerging Millimeter Wave technology [21], or even the optical method including Laser [18] or Infrared Ray [22], etc.
(3) Moreover, the propagation time can also be adopted for detecting the RFT on the LOSL, since the time delay will be increased with the multi-path signal caused by the shadowing of the obstacle, which of course has a harsh requirement on the synchronization accuracy.

Here, we conduct a practical experiment to verify the effectiveness of the spatial diversity technique on RFT detection.

### 3.1. Experiment Environments

**Figure 1:**
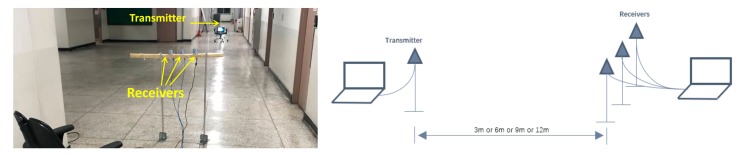
Environment and sketch of RFT detection experiments.

To verify the effectiveness of the diversity reception techniques for RFT detection, 4 XBEE S2C Modules working in 2.4 GHz are adopted, among which one is set as the transmitter, and the receiver consists of three modules. We performed the experiment at an indoor corridor at a general office building of our university under multipath environments, and the experiment sketch is shown as Figure 1. As shown in Figure 1, three receiver antennas are spatially separated, and we set the interval as the radio carrier wavelength, 0.125 m, to meet the requirement of space diversity reception.

### 3.2. RSS Fusion under Spatial Diversity Receiving

**Figure 2:**
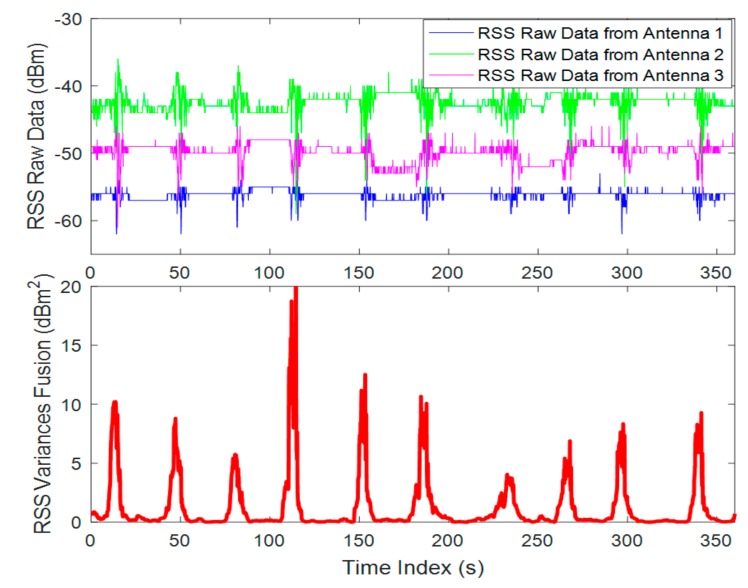
Diversity RSS fusion for RFT identification (RSS sampling rate: 10 Hz).

In general, the RFT means the RSS fluctuation of the LOSL caused by the passing-by target, and it is shown up as the increase of the RSS variances during the specific time-period. Thus, we synchronously calculate the RSS variances of the three modules RSSI values in dBm in a sliding window with a width of M, i.e., ${\mathrm{RSS}}_{n}{|}_{t-M}^{t}$, and then fuse them using the following equation:

(1) $$\mathrm{Fusion}\left(t\right)=\sum_{n=1}^{3}\omega_{n}\left(t\right)\cdot \mathrm{var}\left({\mathrm{RSS}}_{n}{|}_{t-M}^{t}\right)$$

in which M is the width of the sliding window, ${\mathrm{RSS}}_{n}$ is the raw RSS data measured by the n-th antenna, and $\omega_{n}\left(t\right)$ is the normalized weight, which is time-varying and decided by

(2) $$\omega_{n}\left(t\right)=PR_{n}\left(t\right)/\sum_{n=1}^{3}PR_{n}\left(t\right)$$

in which $PR_{n}$ is the RSS mean value in the sliding window, and its unit is Watt, which is calculated by $dBm=10\log\left(\frac{W}{1mW}\right)$. The Equation (2) means that we assign more confidence on the fluctuation that occurred on the antenna with higher RSS level. When the distance between transceivers is 10 m and M=40, a pedestrian traverses the LOSL 10 times at normal speed (0.7 m/s), and the diversity RSS fusion data is presented in Figure 2. As shown in the figure, single receive antenna can detect 10 times of traverses, and detection can be even enhanced with the diversity RSS fusion.

The RFT experimental result shown in Figure 2 indicates that there is an obvious instant increase of the RSS variance on the LOSL when a moving target is passing by; specifically, we can easily detect the LOSL triggering when the device-free target is traversing, by using RFT phenomenon. This confirms the feasibility of applying the RFT phenomenon on passive tracking purpose in the WSNs operating at 2.4 GHz. 

### 3.3. The Influencing Factors of RFT Detection

However, it is currently difficult to guarantee that the RFT phenomenon on the LOSL can be detected by 100 percent under all kinds of radio frequency environments. Several constraints should be noticed for feasible implementation.

The first issue is the operating frequency of the WSN. Actually, the LOSL is the approximation of the first Fresnel zone, which is an elongated ellipsoid, and the obstacle in it can easily affect the RSS [6]. According to the theory of Fresnel zone, the radius of the first Fresnel zone at point P is expressed as

(3) $$R=\frac{1}{2}\sqrt{\frac{d_{1}d_{2}c}{\left(d_{1}+d_{2}\right)f}}$$

in which c is the speed of light, $d_{1}$ and $d_{2}$ are the distances of P from the two wireless nodes, and f is the frequency of the carrier wave. From (3), we can figure out that the approximation mentioned above will be enhanced by wireless signal with higher frequency, and the enhancement will obviously benefit the accuracy of LOSL triggering detection.

The speed of the traversing target is an empirical factor affecting the RFT detection. Assuming that the target is moving at faster speed and the RSS sampling rate is not high enough, we will fail to recognize the RFT detection. As shown in Figure 2, the RFT detection is quite robust, when the target is moving in 0.7 m/s and RSS sampling rate is 10 Hz. If the target is running, this robustness can be still maintained by increasing the RSS sampling rate.

Actually, it is feasible to further guarantee the RFT detection by combining the spatial diversity technique with frequency diversity and the utilization of higher frequency waves; hence, in this paper, we prefer to focus on our passive tracking algorithm, assuming that the RFT phenomenon on all the LOSLs can be perfectly detected once the target passes through.

## 4. Passive Tracking System Description 

### 4.1. The CP Estimation for a Single Target Adopting the Geometric Constraint Method

The geometric constraint method for passive tracking work utilizing RFT phenomenon is briefly presented as follows: Once the wireless nodes are deployed in the WSN area with known and fixed coordinates, the area is partitioned by the LOSLs into regions whose boundaries are the segments on LOSLs (formulas of the LOSLs, as well as their intersections, are all known information, due to the known coordinates of the nodes). When a target moves in the area of interest and traverses the LOSLs, the triggering sequence of the LOSLs is recorded by the system. With the known networking topology information and the triggering sequence, the tracking work based on geometric constraint method is performed to decide in which segment the target is located; then, the middle point of the segment with highest probability is estimated as the CP to minimize the mean-square-error of CP estimation. Finally, the CPs reveal the target’s trajectory. A centralized entity is put to receive the LOSL triggering information from the wireless nodes, and it takes charge of the LOSL triggering sequence storing, analyzing the sequence to determine the CPs; distinguish the behaviors of device-free targets and the requirements of tracking accuracy; and, accordingly, adjust the density of active nodes in the WSN.

**Figure 3:**
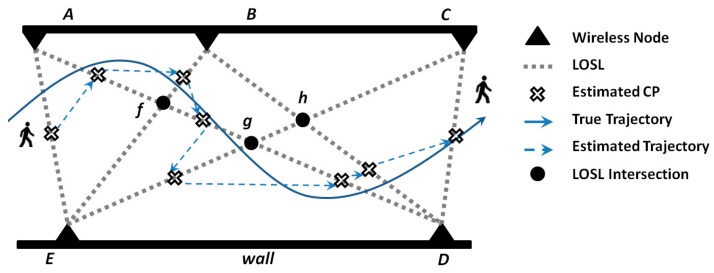
A scenario for the CP estimation of the LOSLs according to triggering sequence and time period.

A typical trajectory identification scenario is shown in the Figure 3 for illustrating the tracking scheme based on geometric constraint in detail. The LOSLs among five wireless sensors (*A*, *B*, *C*, *D,* and *E*), which form a LOSL web, monitoring a corridor. The wireless nodes are fixed with known coordinates as $\left[x_{A},y_{A}\right]$, $\left[x_{B},y_{B}\right]$, $\left[x_{C},y_{C}\right]$, $\left[x_{D},y_{D}\right],$ and $\left[x_{E},y_{E}\right]$. In this light, the formulas of the LOSLs are also known, and the intersection of these LOSL are also thus predetermined as f=L(A,D)∩L(B,E), g=L(A, D)∩L(C, E), and h=L(B, D)∩L(C, E), respectively. If the target is walking across the monitoring area along the trajectory presented in solid line, the triggered sequence of LOSLs L(i), i=1,2,…, 8 will be recorded in order as L(A, E), L(A, D), L(B, E), L(A, D), L(C, E), L(A, D), L(B, D), and L(C, D).

When the first LOSL, L(A, E), is detected to be triggered, the first CP will be simply decided as the middle point of S(A,E) by

(4) $$\left[x_{\mathrm{CP}1},\;y_{\mathrm{CP}1}\right]=\left[\left(x_{A}+x_{E}\right),\left(y_{A}+y_{E}\right)\right]/2,$$

since just one segment S(A,E) is located on the LOSL. After passing the L(A, E), the target walks into the region, R(1)=R(A,E,f).

The second triggered LOSL is recorded as L(A, D), which is divided into three segments by the other LOSLs: S(A,f), S(D,g), and S(f,g). The probability of the CP on S(f,g) will be zero, because the target comes from the region R(A,E,f), but L(B,E) is not triggered before L(A, D). Similarly, it is more impossible that the CP is located at S(g,D), since L(B, E) and L(C, E) are not triggered at all. Thus, we decided that the CP is located at S(A,f). After passing the S(A,f), the target gets in the region R(2)=R(A,B,f). 

The third triggered L(3) is L(B, E). Around the region R(A,B,f), the nearest segment on L(B, E) is S(B,f); on the other hand, it is impossible that the target can reach S(E,f) without triggering L(A,D) again. Under this light, the third CP is most likely located on S(B,f), so the middle point of S(B,f) is selected as the third CP, after passing S(B,f); the target walks into R(3)=R(B,f,g,h) with highest probability. After new LOSLs are detected to be triggered, the CPs are marked by the empty crosses, as shown in the figure. 

Generally, the framework of proposed CP estimation for the single target can be briefly summarized as Algorithm 1:
**Algorithm 1. The CP Determination for Tracking a Single Target****Initialization:** Get information of the WSN topology, including the node positions, LOSL formulas **Step 1:** get the first CP as the middle point of the edge LOSL L(1), and decide R(1). **Step 2:** Once L(i), i>1 is triggered  Select the highest probability segment S(i) on L(i), which is nearest to R(i−1).  Estimate CP as the middle point of S(i) and decide that R(i) is the opposite region of S(i). **Step 3:** Go back to **Step 2**, until the target walks out of the monitoring area.

### 4.2. The Approach for Multiple Targets Tracking

If two or more targets are walking in the web, the problem becomes multiple targets tracking problem. Here, we present the divide-and-conquer approach to identify the trajectories of two device-free targets, although the maximal number of targets that can be simultaneously tracked is more than two and can be further extended by increasing the density of wireless nodes.

**Figure 4:**
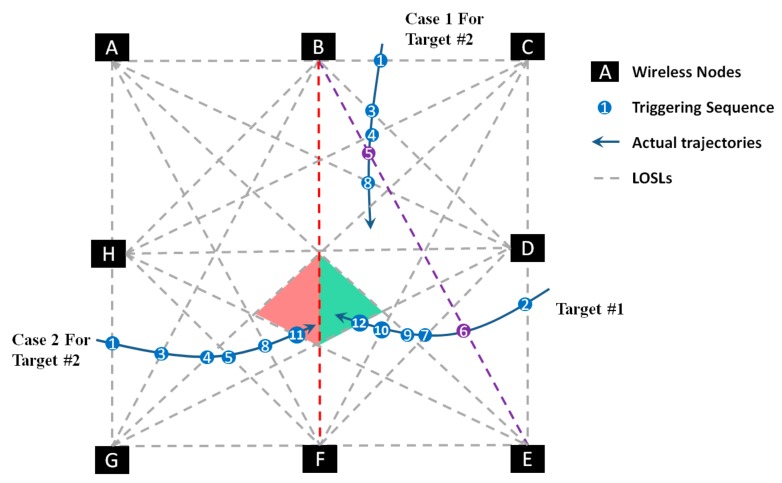
Scenarios for two cases of two targets. For the first case, it is possible to uniquely distinguish the two trajectories separately, while predicaments will happen in the second case.

Two cases for tracking two targets are presented as an example in Figure 4. Firstly, we discuss Case 1: If the two targets, including Target #1 and Target #2, are walking in the monitoring area according to the Case 1, as illustrated in Figure 4, the triggering sequence is L(i), i=1,2,…, 9, which is recorded as L(B,C), L(D,E), L(B,D), L(C,H), L(B,E), L(B, E), L(D, F), L(A, D), and L(A, E). Firstly, from the measurement, we can easily conclude that there are two targets in the monitoring zone, because L(B, C) and L(D,E) are impossible to be triggered successively by one single target from the known topology knowledge of wireless node distribution. Then, we assume that Target #1 has walked into the area from L(D,E), and Target #2 has entered from L(B,C) end point, respectively. When L(B, D) is triggered as L(3), the system will deem that L(B, D) is triggered by Target #2, because the L(B, E) is not one of the sides of the region where Target #1 is currently located. For similar reasons, we can deduce that Target #2 has triggered L(C,H) as L(4). Then, the targets can be tracked by Algorithm 1 separately. 

When the WSN system yields data that L(B,E) is triggered twice as L(5) and L(6), the situation is incomprehensible, because L(B,E) is the straight line containing the sides of both regions where Target #1 and Target #2 are currently located. There are multiple probabilities to consider, and there is no way to distinguish between these probabilities to figure out the original trajectory. The probable explanations are as follows:

Explanation 1: L(B,E) is triggered by only Target #2 twice;
Explanation 2: L(B,E) is triggered by only Target #1 twice;
Explanation 3: L(B,E) is triggered by Target #2 as L(5) and by Target #1 as L(6);
Explanation 4: L(B,E) is triggered by Target #1 as L(6) and by Target #2 as L(5).

So, we move on to the next step and collect further data from the system on the next triggers in LOSLs. From the following measurements, we can see that L(D,F) is triggered as L(7). It is reasonable for all the four explanations to trigger L(D,F) as L(7), and we cannot eliminate any explanations by this measurement; thus, we need to get further measurements. In further measurements, L(A,D) is triggered as L(8), and L(A,E) is triggered as L(9). Now, it is possible for us to add this measurement to analyze and eliminate the probabilities one by one until we get the unique trajectories.

**Figure 5:**
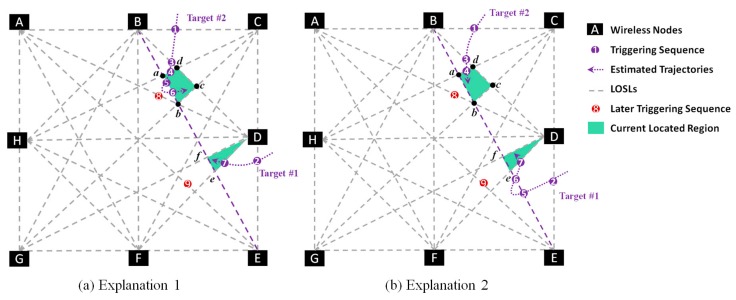
Illustration for eliminating the fake Explanation 1 and 2 using the later triggering sequence information.

From the prior topology knowledge shown in Figure 5a, we can calculate that if L(B,E) was triggered only by Target #2 as L(5) and L(6), then the target will be located in R(a,b,c,d). Since the L(7) is L(D,F), it must be triggered by Target #1, and thus Target #2 is located at R(D,e,f). Afterwards, L(A,D) is triggered as L(8), and L(A,E) is triggered as L(9). It is impossible to get the next trigger in L(A, D) or L(A, E) considering the current location of the two targets, because neither L(A, D) or L(A, E) are sides of R(D,e,f) and R(a,b,c,d). Hence, Explanation 1 is eliminated. In the same way, Explanation 2 in Figure 5b can be also eliminated using the measurements of later triggering sequences L(8) and L(9). 

So, we can determine that the triggers on L(B,E) were made by both Target #2 and the Target #1. However, it is impossible to decide which trigger was made first. By applying geometric constraints, we can easily deduct that the trigger on L(A,D) was made by Target #1 and the trigger on L(A, E) was made by Target #2. Thus, we can complete the trajectory of two separate targets without determining which of the targets made the triggers at L(8) and L(9). Moreover, the failure in solving the predicament of the trigger timing between L(5) and L(6) does not affect the overall trajectory of the targets. 

As discussed above, when the targets are located in separate areas in the monitoring area and they trigger the same LOSL, it is possible to calculate the trajectories by considering the later LOSL triggering sequence and eliminating the impossible possibilities. Moreover, we deduct the trajectory by step-wise calculation and get the final result which that fits in and explains all other criteria. However, when the targets are in close proximity to each other, it is sometimes impossible to find the exact trajectories. 

For an example, in Case 2 of Figure 4, by measuring the data provided by the system and Algorithm 1, we can figure out that Target #1 is currently located in the aqua region, and Target #2 happens to enter one of the neighboring regions of Target #1 (the region in pink). Under this light, if L(B, F) is triggered as L(13), it would be mathematically impossible to figure out which of the targets triggered L(B, F), but we know that the two device-free targets are in the same region. When the targets are in neighboring regions, the system may fail to determine the precise trajectory uniquely, which we call the “meeting event”. Meanwhile, it is pretty clear that, with an increased density of LOSLs, the probability of the targets ending up in the neighboring region will become lower.

In general, the approach for tracking the multi-targets can be briefly presented by Algorithm 2:
**Algorithm 2. The CP Determination for Tracking Multiple Targets.****Initialization:** Get the coordinates of all wireless sensor nodes, as well as topology information of all LOSLs. **Step 1.** Decide the number of targets by triggering LOSL in separated zones  **if** the targets do not meet at the neighbor regions.    **Step 2.**Divide the triggering LOSLs into groups belonging to different targets.   **Step 3.** Individually perform tracking work on each target by using the geometric constraint method presented in **Algorithm 1**;   **Step 4.** List all the possible explanations when multiple targets are triggering the same LOSL and conflict occurs.   **Step 5.** Further adopt the later trigging LOSL measurements to eliminate the other possible explanations to get the unique solution.
  **else**    Give warning sign and increase the LOSL density to solve the meeting event.  **end**

## 5. The Power Consumption Model

In wireless communication scenarios, the RSS can be measured only when the wireless node pairs are undergoing communication, which will result in power consumption. Under the ideal wireless propagation model, the relationship between RSS $P_{r}$ and transmitting power $P_{t}$ in Watt in terms of distance d can be expressed as follows [20]:

(5) $$P_{r}\left(d\right)=\frac{P_{t}G_{t}G_{r}\lambda^{2}}{{\left(4\pi \right)}^{2}d^{2}\gamma }$$

in which $G_{t}$ and $G_{r}$ are the transmitter and receiver antenna gains, respectively; λ is the carrier wavelength; and γ is the constant system loss factor. If the power is presented in units of dBm, the above model can be represented as follows:

(6) $$P_{R}\left(d\right)=P_{T}-\;20\mathrm{lg}d+\phi +X_{n},$$

in which $P_{T}$ and $P_{R}$ are the transmitting and the receiving power in dBm, respectively; ϕ is a constant system parameter, decided by $\log\frac{G_{t}G_{r}\lambda^{2}}{{\left(4\pi \right)}^{2}\gamma }$; and $X_{n}$ is a random deviation variable with zero mean, following the Normal distributions. 

If the minimum receiving power is fixed as $P_{RFT}$ for guaranteeing the LOSL triggering detection, then the required transmitting power $P_{T}^{\prime }\left(d\right)$ should satisfy the following condition:

(7) $$P_{T}^{\prime }\left(d\right)>P_{RFT}+\;20\mathrm{lg}d-\phi$$

**Figure 6:**
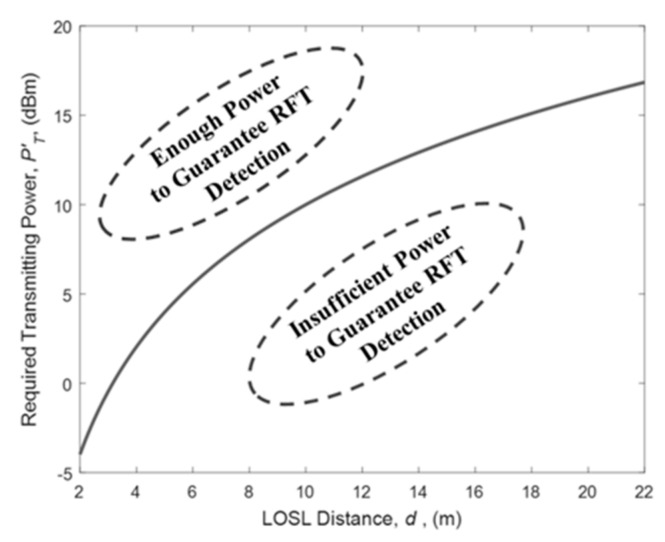
Required power for the transmitter to guarantee RFT detection in terms of the LOSL distance.

In a WSN operating at 2.4 GHz, ϕ and $P_{RFT}$ can be empirically set as −40 dBm and −60 dBm, respectively. Under this light, Figure 6 shows the condition of the minimum transmitting power with this model. As shown in the figure, the transmitting power has to be on the upper side of the solid line for RFT identification.

Suppose the WSN contains K active nodes forming a LOSL web containing $\frac{K\left(K-1\right)}{2}$ LOSLs, the power consumption in Watt of this network would be expressed as follows:

(8) $$\sum_{j=i+1}^{K}\sum_{i=1}^{K-1}10^{10^{-3}\cdot P_{T}^{\prime }\left(d_{i,j}\right)},\;i,\;j\in \left[1,\ldots ,K\right]\;\mathrm{and}\;i<j,$$

in which $d_{i,j}$ is the LOSL distance, i.e., the length of the LOSL between i-th and j-th nodes.

## 6. The Adaptive Solution for Energy Conservation

In the CP estimation scheme mentioned in Section 4, it is apparent that higher LOSL density can refine passive tracking performance, since more LOSLs can cut the individual LOSL into smaller segments. As a matter of fact, in common WSNs, the topology control mechanism should be adopted to provide energy efficiency and to extend network lifetime by turning off the nodes that are redundant. Hence, it is not energy-efficient to specifically maintain a complex web of communication links undergoing unnecessary signal transmission just for the purpose of target tracking. In this context, therefore, we propose an adaptive wireless scheme to guarantee the passive tracking performance while addressing energy conservation.

**Figure 7:**
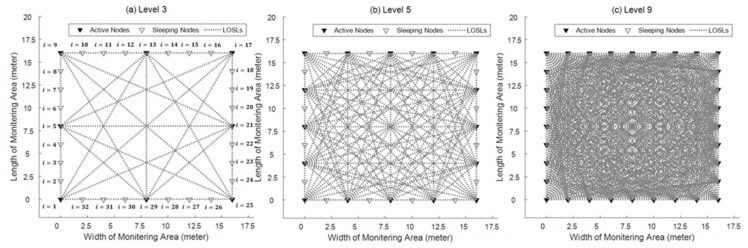
Three topical levels of the proposed adjustable networking scheme (N = 3, 5, 9).

Figure 7 shows a WSN scenario in which the proposed adaptive wireless networking scheme can be performed; a similar scenario is widely adopted by the RTI-based and LOSL-based passive tracking schemes. There are 32 wireless nodes in total monitoring a 16-by-16 m^2^ area. For energy conservation, not all of the nodes are communicating with other nodes; instead, we just choose parts of them to form a LOSL web. For example, in Figure 7a, only 8 of the 32 nodes are chosen as active nodes, while the others stay in sleeping mode; we define this situation as Level 3, since there are 3 active nodes on one side of the rectangular area; in the same way, Figure 7b,c corresponds to Level 5 and Level 9, respectively.

Here, we provide a level classification approach for the popular experimental scenarios in Figure 7. In Level N, we selected the node set $K_{N}$ including 4·(N−1) nodes, which can be denoted as follows:

(9) $$K_{N}=\left\{k_{1},k_{2},\ldots ,k_{4\cdot \left(N-1\right)}\right\}\;k_{i}=1+\langle \frac{8\cdot \left(i-1\right)}{N-1}\rangle ,$$

in which 〈*〉 is the symbol of round function. The constant 8 is derived from dividing the total node number 32 by the four sides.

In the proposed scheme, the WSN is initialized by remaining in the lowest Level 3, as the “standby state” with a minimum number of LOSLs, where it waits for the target. When the target gets into the monitored area, the level of LOSL density will be dynamically adjusted depending on the situations for the purpose of energy conservation while guaranteeing the tracking performance.

The system adaptively increases the LOSL density level under two representative situations: when we want to get better tracking accuracy of the target, or when the trajectories of targets are not clear enough. As for the first situation, if we just want to get higher accuracy, we can make it by increasing the number of LOSLs between node pairs. By changing WSN from Level 3 to Level 5, then, we can get more CPs, and we can track the trajectory of the target more accurately. The second scenario specifically means that the meeting event happens under multi-target scenario, or abnormal LOSL sequence emerges; then, we can assume that there will be a tracking error. The tracking error is not easily corrected with only the low level of web. On the contrary, for energy saving, the system will automatically put some of the nodes into sleep mode when the tracking accuracy requirement is lowered. This action will also be performed when no indecipherable LOSL sequence emerges within a predetermined time period.

**Figure 8:**
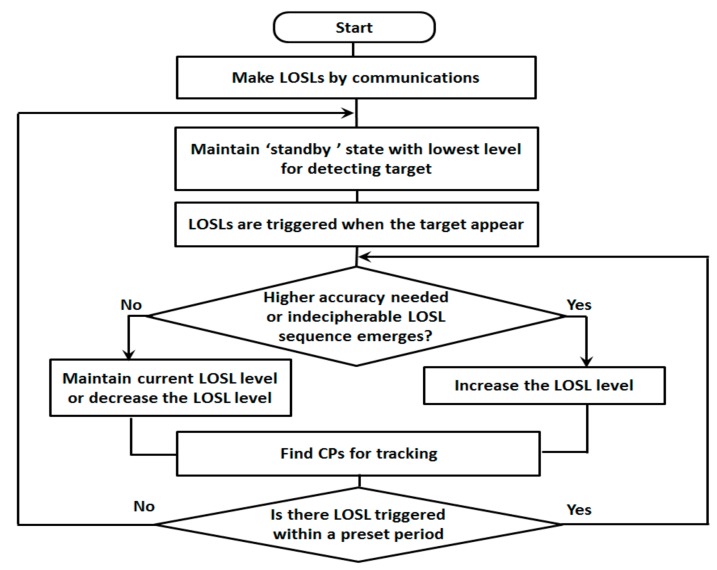
Flow chart of proposed adaptive networking scheme for tracking device-free targets.

Figure 8 is a brief flow chart of the proposed adaptive scheme. For the first step, the LOSLs for target detection maintain “standby state” with only minimum basic links for detecting target while saving energy. When a target is detected with the basic links, the proposed scheme increases the complexity of web with more LOSLs. Meanwhile, the proposed scheme checks the accuracy requirement, as well as the appearance of meeting events, to determine whether it maintains the current level of LOSL, or decreases the LOSL density for energy saving, or it gets higher level of LOSL for enhancing the tracking accuracy. The meeting events can be considered as a sequence error and an error of missing LOSL, which can affect the tracking accuracy. In the next step, ask again and repeat the former step’s process, and if there are no more events, move to the final step and make the crossing points for tracking work. When no LOSLs are triggered for a while, the system can figure out that the target has already moved out of the monitoring region and get back to standby state.

## 7. Performance Evaluation

### 7.1. The Tracking Performance Evaluation of the Proposed Passive Tracking Method

After acquiring the prior knowledge of the RFT identification through practical experiment in Section 3, the proposed scheme was evaluated with computer simulations. We assume 32 wireless nodes are adopted to monitor a 16×16 m^2^ area. The level of the adaptive network ranges from Level 2 to Level 9 according to the adapting node number in Table 1. When a target walks across the monitoring space with a random trajectory, the LOSL triggering sequence L(i) is recorded by the system, and the CP on each individual LOSL is estimated using the approaches presented in Section 4.1. Meanwhile, the power consumption under each level is worked out by Equation (8) in Section 5.

**Figure 9:**
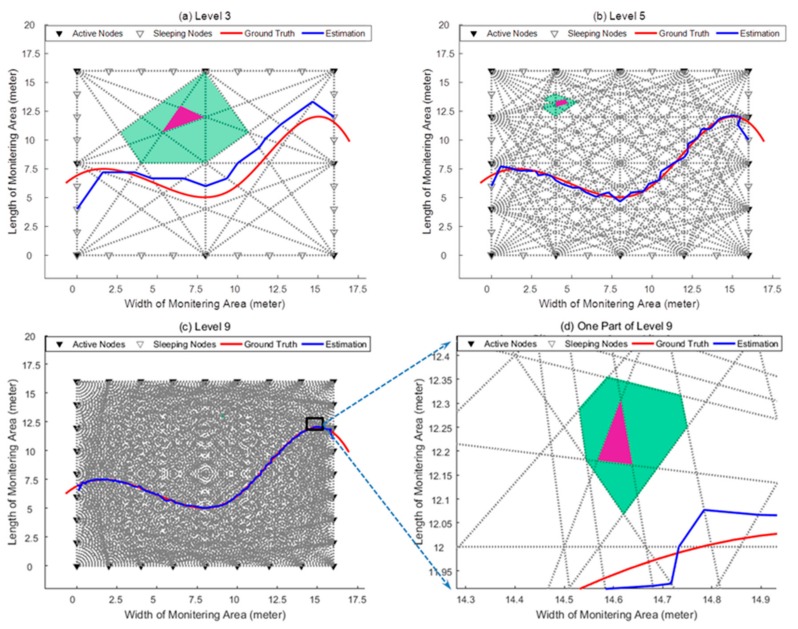
Tracking results under different levels.

**Figure 10:**
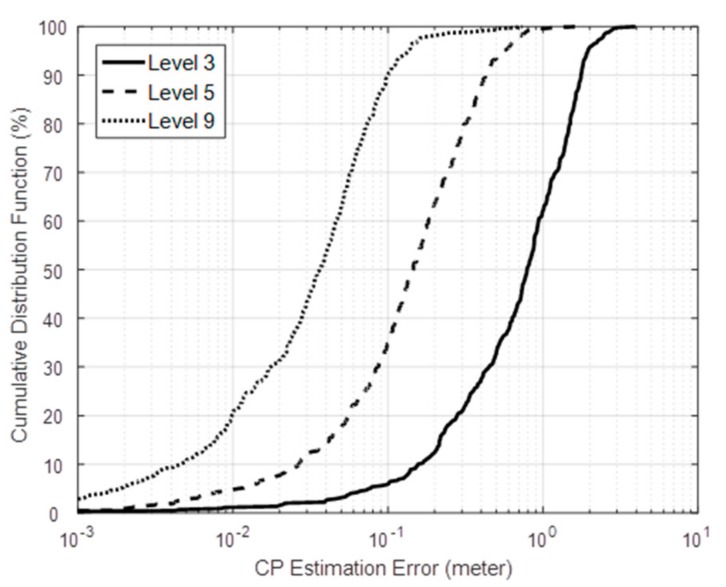
Error statistics under Levels 3, 5 and 9.

Results of the proposed scheme based on the geometric constraint method are shown in Figure 9: The actual trajectory of the target is made randomly. After identifying the CPs on the LOSLs, their connection indicates the estimated trajectory in blue line. It can be seen from Figure 9a–c that the more active nodes are adopted, the higher LOSL density being distributed, and, as a result, the more closely the estimated trajectory approximates to the ground truth. Then, we performed 100 Monte Carlo runs under Levels 3, 5 and 9; the error cumulative distribution function (CDF) is given in Figure 10. Under Level 3 with low hardware cost, more than 60 percent of the error is below 1 meter; what should be noted is that 90 percent of the simulation tracking error is below 0.1 m at the highest level (Level 9), which is a really efficient performance considering that the nodes are placed at intervals of 2 m.

Our proposed method is compared with existing RTI, as well as the NOOLR method in Table 2. The node density is computed as the quotient of the adopted node number and monitoring area. We can see that the passive tracking scheme based on the geometric constraint methodology outperforms the other two existing methods, even though it is implemented under lower node density.

### 7.2. The Performance Evaluation of Multi-Target Tracking and Energy Conservation

As we discussed in the multiple target scenario, the targets can be tracked using the single target tracking method and the subsequent LOSL triggering measurements when they are moving in separated regions. However, when the targets meet at the neighbor regions, the estimation work for multiple trajectories can have manifold forms of solution, which results in tracking failure. Increasing the web level can mitigate this problem. Figure 9 clearly shows the area size of the conflict regions (the surrounding aqua zone) of one specific region (the central magenta zone). It is apparent that the conflict region is shrunken as the LOSLs density increases (more than 10 m^2^ at Level 3, around several square meters at Level 5, and less than 1 m^2^ at Level 9, see in Figure 9d), which means that the more nodes are activated, the lower the probability becomes of the targets meeting at the conflict zone, and the lower the probability of tracking failure there will be; meanwhile, the maximal number of targets that can be simultaneously tracked will be extended.

**Figure 11:**
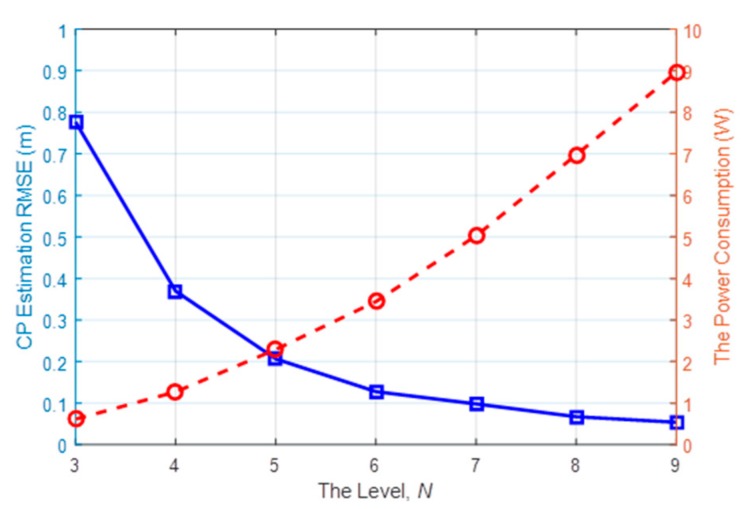
Root-mean-square error (RMSE) and the power consumption at each level.

After 100 independent Monte Carlo runs, Figure 11 presents the root-mean-square error (RMSE) of the CP estimation from the Level 3 to 9, as well as the power consumption. Unsurprisingly, the tracking performance is improved by increasing the number of active nodes. What should be noted is that when the level is higher than 5, the CP estimation RMSE can be suppressed below 0.2 m. However, the enhancement in terms of active node number tends to be stolid at higher levels, while the power consumption (shown as the red dashed line) is steadily rising when more nodes are activated for communication to form the LOSLs. This suggests that the adaptive network undergoing tracking work can be set at Level 5 by default, and then the LOSL density can be dramatically adjusted for the consideration of energy conservation and tracking performance.

**Figure 12:**
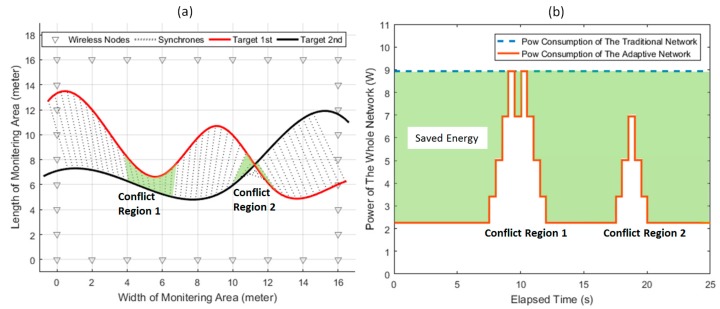
(**a**) A specific multitarget tracking scenario in the WSN. (**b**) The energy saving in the adaptive network, relative to the traditional network.

To show the energy efficiency of the adaptive networking scheme, simulations are conducted as follows. Assuming that two device-free targets are passing through the monitoring area simultaneously from left to right, an adaptive networking scenario is illustrated in Figure 12a. The synchrones represented by the dotted lines represent the relative positions of the two targets with respect of time. In the network, we assume that the required accuracy can be realized by Level 5, and Level 9 should be maintained to prevent the meeting event when the targets are getting close. 

In the traditional network for tracking, the networking level has to be constantly set as 9 to guarantee multi-target tracking success, whereas the adaptive network can adjust the networking level in real time. As shown in Figure 12b, the level of the adaptive network can instantly be increased by the system when the targets are close for mitigating the meeting event (the two targets are close enough to arouse level upgrade in Conflict Region 1 and Conflict Region 2 marked in the figure) and can be decreased when no meeting conflict happens. This is obviously more power efficient, and the energy efficiency is quantified as the light-colored area in Figure 12b. Numerical computation indicates that the energy usage of above case can be decreased by 63.8% (energy consumption of traditional scheme is 224 Joule, while that of the adaptive scheme is 81.1 Joule), which clearly illustrates the necessity and effectiveness of the adaptive networking mechanism for passive tracking in WSNs.

## 8. Conclusions and Future Works

In this paper, we proposed a passive tracking scheme exploiting adaptive networking LOSL webs. In the proposed scheme, the LOSL density level between wireless nodes pairs can be constructed flexibly to address the energy conservation while guaranteeing the tracking performance. Firstly, as fundamental knowledge, we explore the feasibility of applying the RFT for moving target tracking. For the quantifiable performance evaluation of the proposed tracking scheme, we mathematically presented the CP estimation under both single-target and multi-target scenarios, as well as the power consumption model in a specific WSN environment. The simulation results show that the proposed passive tracking method based on CP estimation can produce rather high accuracy with lower hardware-cost; moreover, the power consumption of the adaptive networking scheme in a specific tracking scenario can decrease by around 60 percent.

As for the future work, the effectiveness verification of the proposed tracking scheme in realistic experiments is the main task for the moment. The biggest challenge is improving the effectiveness of RFT detection, because the passive tracking scheme will be out of question if we have no accurate measurement of the LOSL triggering. Although we have demonstrated that the RFT on the LOSL can be detected in the Section 3, however, the RFT detection under more complex indoor environments with various obstacles will be an intractable challenge. Another issue that should be investigated is improving the detection accuracy of the LOSL triggering time. Our measurement is the LOSL triggering sequence instead of the accurate triggering timestamp, which implies that there naturally exists a fault-tolerant ability for triggering time deviation; however, in networks with dense LOSLs, an accurate triggering-time detection algorithm or a fault-tolerant mechanism for the trigger sequence error should be researched to enhance the feasibility of the passive tracking scheme based on the geometric constraint method.

## Figures and Tables

**Table 1:** Level classification of the WSN with 32 nodes.

Level *N*	Number of Active Nodes	Nodes Set
3	8	$K_{3}=\left\{1,5,9,13,17,21,25,29\right\}$
4	12	$K_{4}=\left\{1,\;4,\;6,\;9,\;12,\;14,\;17,\;20,\;22,\;25,\;28,\;30\right\}$
5	16	$K_{5}=\left\{1,\;3,\;5,\;7,\;9,\;11,\;13,\;15,\;17,\;19,\;21,\;23,\;25,\;27,\;29,\;31\right\}$
6	20	$K_{6}=\left\{1,\;3,\;4,\;6,\;7,\;9,\;11,\;12,\;14,\;15,\;17,\;19,\;20,\;22,\;23,\;25,\;27,\;28,\;30,\;31\right\}$
7	24	$K_{7}=\left\{1,\;2,\;4,\;5,\;6,\;8,\;9,\;10,\;12,\;13,\;14,\;16,\;17,\;18,\;20,\;21,\;22,\;24,\;25,\;26,\;28,\;29,\;30,\;32\right\}$
8	28	$K_{8}=\left\{1,\;2,\;3,\;4,\;6,\;7,\;8,\;9,\;10,\;11,\;12,\;14,\;15,\;16,\;17,\;18,\;19,\;20,\;22,\;23,\;24,\;25,\;26,\;27,\;28,\;30,\;31,\;32\right\}$
9	32	$K_{9}=\left\{1–32\right\}$

**Table 2:** Passive tracking performance comparison.

Tracking Scheme	Hardware Cost ^①^	Variance	Mean Error
Proposed Method (Level 5)	0.0625 nodes/m^2^	0.035 m^2^	0.199 m
NOOLR Method	0.0726 nodes/m^2^	0.037 m^2^	0.214 m
RTI Method	0.0726 nodes/m^2^	0.130 m^2^	0.251 m

^①^ Note that the performance data of NOOLR and RTI are quoted from the Ref. [16], in which the node interval is 3 m, monitored by 21-by-21 m^2^ area, while Level 5 of proposed method is implemented in a 16-by-16 m^2^ area when the node interval is 4 m.
